# Cell-based immunotherapies for solid tumors: advances, challenges, and future directions

**DOI:** 10.3389/fonc.2025.1551583

**Published:** 2025-04-28

**Authors:** Ting Zhao, Jinping You, Congyue Wang, Bo Li, Yuhan Liu, Mingjia Shao, Wuyang Zhao, Chuang Zhou

**Affiliations:** ^1^ Department of Oncology, Ansteel Group General Hospital, Anshan, China; ^2^ Department of Medical Oncology, Anshan Cancer Hospital, Anshan, China

**Keywords:** cell-based immunotherapy, CAR-T, CAR-NK, TCR-T, solid tumors, tumor microenvironment

## Abstract

Cell-based immunotherapies, including CAR-T, CAR-NK, and TCR-T therapies, represent a transformative approach to cancer treatment by offering precise targeting of tumor cells. Despite their success in hematologic malignancies, these therapies encounter significant challenges in treating solid tumors, such as antigen heterogeneity, immunosuppressive tumor microenvironments, limited cellular infiltration, off-target toxicity, and difficulties in manufacturing scalability. CAR-T cells have demonstrated exceptional efficacy in blood cancers but face obstacles in solid tumors, whereas CAR-NK cells offer reduced graft-versus-host disease but encounter similar barriers. TCR-T cells expand the range of treatable cancers by targeting intracellular antigens but require meticulous antigen selection to prevent off-target effects. Alternative therapies like TIL, NK, and CIK cells show promise but require further optimization to enhance persistence and overcome immunosuppressive barriers. Manufacturing complexity, high costs, and ensuring safety and efficacy remain critical challenges. Future advancements in gene editing, multi-antigen targeting, synthetic biology, off-the-shelf products, and personalized medicine hold the potential to address these issues and expand the use of cell-based therapies. Continued research and innovation are essential to improving safety, efficacy, and scalability, ultimately leading to better patient outcomes.

## 1 Introduction

Cancer remains one of the most formidable challenges in modern medicine, with conventional treatments such as radiation, chemotherapy, and surgery often hindered by issues like lack of personalized approaches, significant adverse reactions, tumor heterogeneity, and the development of drug resistance (1, 2). Cell therapy, tracing its origins to the 19th century, has significantly evolved from the initial injections of animal materials to sophisticated human cell-based treatments, most notably bone marrow transplants (3). Immunotherapy, a subset of cell therapy, enhances the body’s immune system to recognize and combat cancer cells, showing remarkable promise across various cancer types (4).

**Figure 1:**
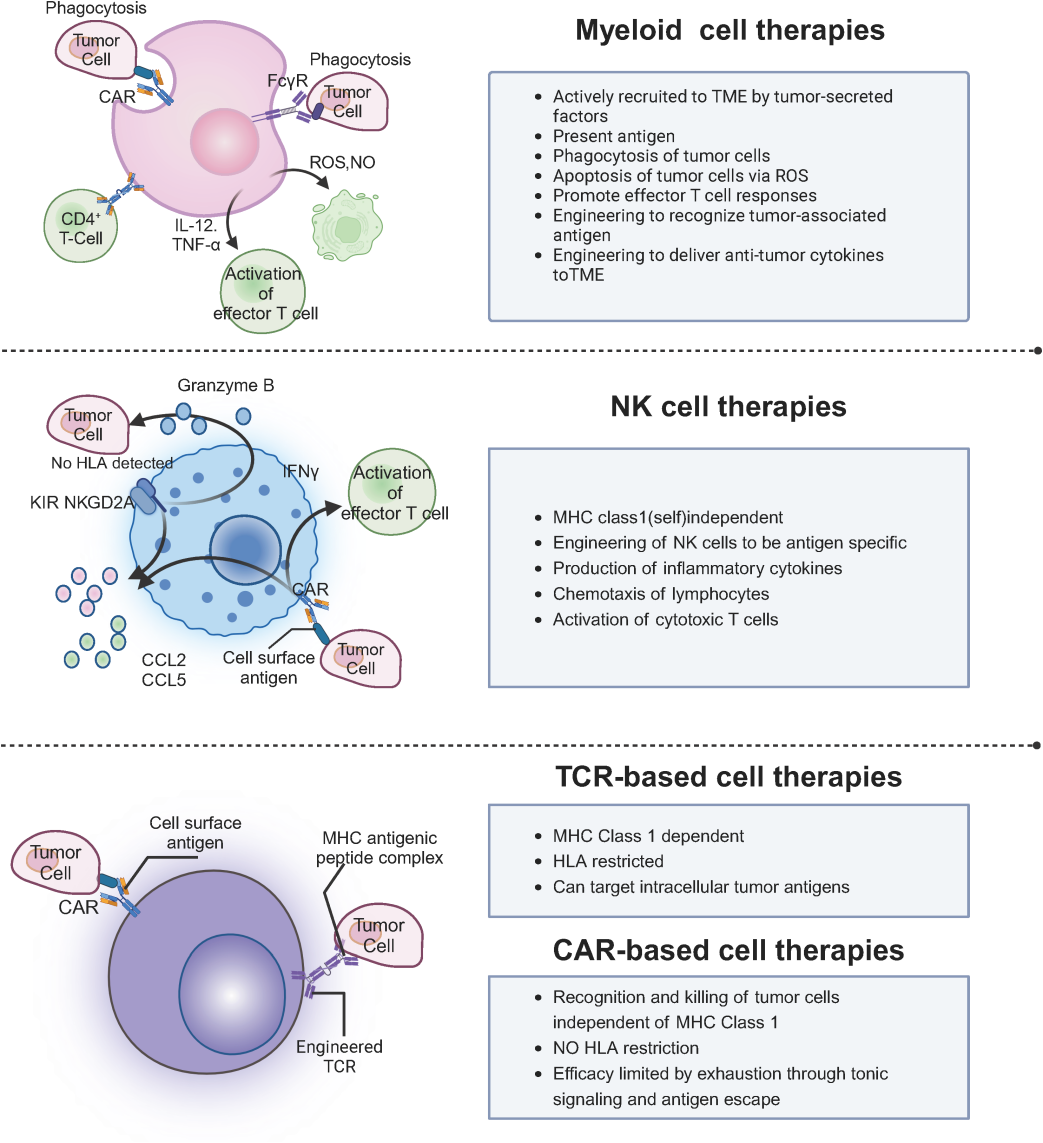
Mechanisms of myeloid, NK, TCR, and CAR-based cell therapies in TME.

Key immune cells, including lymphocytes, macrophages, and cytotoxic T cells, target tumor-specific antigens, driving the development of therapies such as granulocyte colony-stimulating factor, interferons, and investigational agents like IL-2 and chemokines, which are advancing through clinical trials (5, 6). While cell therapy offers precise tumor targeting, immune system enhancement, and potential long-term anticancer effects, it also faces challenges such as drug resistance, immune suppression, adverse reactions, and risks like infections (7). Moreover, clinical studies are still in early stages for many therapies, and the complex, costly production processes limit widespread application (8). This manuscript aims to provide a comprehensive overview of cell-based immunotherapies, focusing on CAR-T, CAR-NK, and TCR-T therapies, and discusses the challenges and future prospects in their application to solid tumors, as shown in **Figure 1**.

## 2 CAR-based immunotherapy

CAR-T therapy has revolutionized the treatment of hematologic malignancies, showing remarkable efficacy in diseases like acute lymphoblastic leukemia and certain types of lymphoma (9). However, its translation to solid tumors has proven much more challenging. Similarly, Chimeric Antigen Receptor Natural Killer (CAR-NK) cell therapy has emerged as a promising alternative, leveraging the innate cytotoxic capabilities of NK cells to target cancer cells. Unlike T cells, NK cells offer benefits such as a lower risk of graft-versus-host disease and potentially fewer severe side effects (10). Despite these advantages, CAR-NK therapy also faces significant obstacles, particularly in treating solid tumors. This section explores the primary challenges encountered by both CAR-T and CAR-NK cell therapies in solid tumors, highlights recent studies addressing these issues, and discusses potential solutions currently under investigation.

### 2.1 Antigen heterogeneity and target selection

Solid tumors exhibit significant antigen heterogeneity, complicating the selection of suitable target antigens for CAR-T cells. Unlike hematologic cancers with universally expressed targets like CD19, solid tumors often lack such definitive markers, increasing the risk of antigen escape (10). Recent studies demonstrate that dual-targeting CAR-T cells, engineered to recognize two different antigens, can reduce antigen escape (11). For instance, CAR-T cells targeting both HER2 and IL13Rα2 in glioblastoma have shown improved efficacy (12). Tandem CARs, which incorporate two antigen-binding domains within a single molecule, also enhance the ability to target heterogeneous tumor cells (13). Similarly, solid tumors present antigen heterogeneity challenges for CAR-NK therapy. Dual-targeting CAR-NK cells have been engineered to target multiple antigens, thereby improving therapeutic efficacy and reducing antigen escape. For example, CAR-NK cells targeting both HER2 and EGFR in breast cancer have shown enhanced efficacy compared to single-target cells (14). Bispecific and trispecific CAR-NK cells, and universal CAR platforms, offer flexibility to target different antigens without generating new constructs (15).

CAR structural components impact efficacy, persistence, and safety. The scFv is the antigen-binding domain, defining specificity and affinity (16). High-affinity scFvs improve targeting but may increase off-tumor toxicity. The hinge region connects scFv to the transmembrane domain, influencing flexibility and stability (17). A longer hinge aids clustering but may raise toxicity risk. The transmembrane domain anchors the CAR, affecting stability and expression. Co-stimulatory domains like CD28 and 4-1BB are key for T cell activation and persistence. CD28 boosts initial activation but may cause exhaustion and CRS. 4-1BB supports sustained activation and long-term persistence but may slow early responses. Combining these domains optimizes CAR efficacy and safety (18).

Gene delivery methods are key to the efficiency, safety, and scalability of CAR therapies. Viral vectors (e.g., lentiviruses, retroviruses) provide high transduction efficiency and stable CAR expression but carry risks like insertional mutagenesis and immune responses (19). Lentiviral vectors are preferred for long-term expression, though mutagenesis remains a concern. Non-viral systems (e.g., Sleeping Beauty) offer high transfection efficiency without genome integration, reducing mutagenesis risk, but have lower efficiency than viral vectors, impacting production scale and CAR consistency (20). Electroporation can induce cell stress and reduce viability (21). Balancing efficiency, safety, and scalability is crucial, especially for treating solid tumors.

### 2.2 Tumor microenvironment immunosuppression

TME in solid tumors is highly immunosuppressive, characterized by regulatory T cells (Tregs), myeloid-derived suppressor cells (MDSCs), and inhibitory cytokines like TGF-β and IL-10, which impair CAR-T cell function and persistence (22). Recent studies have shown that armored CAR-T cells, engineered to secrete pro-inflammatory cytokines such as IL-12 or IL-18, can counteract immunosuppressive signals, improving CAR-T cell efficacy (23). Additionally, combining CAR-T therapy with checkpoint inhibitors like anti-PD-1 or anti-PD-L1 antibodies has enhanced CAR-T cell activity within the TME (24). CAR-NK cells also encounter similar immunosuppressive factors in the TME, including Tregs, MDSCs, and inhibitory cytokines (25). To enhance CAR-NK therapy, armored CAR-NK cells have been engineered to secrete pro-inflammatory cytokines like IL-15 or express dominant-negative receptors to counteract immunosuppressive signals in the TME (26). Combining CAR-NK therapy with checkpoint inhibitors has been shown to improve CAR-NK cell activity within the tumor (27). Genetic modifications to produce supportive cytokines, chemokines, or receptors, and to eliminate immunosuppressive cells like Tregs and MDSCs, are being explored to improve CAR-NK cell function (28).

In hematologic malignancies, the TME supports CAR-T cell expansion, activation, and persistence with cytokines like IL-2 and IL-15, which promote T cell proliferation and long-term survival (29). IL-2 enhances T cell expansion, while IL-15 supports memory T cell formation (30). These cytokines enable effective CAR-T responses and better outcomes in hematologic cancers. In contrast, solid tumors have an immunosuppressive TME, dominated by cytokines like TGF-β, IL-10, and IL-4, which hinder CAR-T and CAR-NK function (31). TGF-β promotes regulatory T cells and myeloid-derived suppressor cells, limiting immune responses (32). To overcome this, armored CAR-T and CAR-NK cells are engineered to secrete pro-inflammatory cytokines like IL-12 or IL-18, counteracting TGF-β and IL-10, and enhancing immune activation (33). Combining checkpoint inhibitors, like anti-PD-1/PD-L1, further boosts CAR therapy efficacy in solid tumors.

### 2.3 Limited trafficking and infiltration into solid tumors

Efficient trafficking of CAR-T cells into solid tumors is impeded by physical barriers like the dense extracellular matrix (ECM) and abnormal vasculature, as well as the absence of appropriate chemokine gradients (34). Recent strategies to improve CAR-T cell infiltration include engineering CAR-T cells with chemokine receptors (e.g., CCR2b, CXCR1) to enhance homing and penetration into tumors (35). Additionally, expressing matrix-degrading enzymes like heparanase facilitates the breakdown of ECM components, enabling deeper tumor penetration (36). Localized delivery of CAR-T cells directly to the tumor site can bypass systemic trafficking barriers, increasing efficacy while reducing toxicity (37). Similarly, the infiltration of CAR-NK cells into solid tumors is hindered by physical barriers like the dense ECM and abnormal vasculature. Engineering CAR-NK cells with chemokine receptors like CCR2 or CXCR1 to enhance tumor homing has been shown to improve trafficking (38). The use of matrix-degrading enzymes like heparanase also facilitates deeper penetration into tumors (39). Localized delivery of CAR-NK cells directly to the tumor site can help bypass systemic barriers, increasing the concentration at the target site and reducing toxicities.

### 2.4 On-target, off-tumor toxicity

Many antigens expressed on solid tumors are also present at low levels on normal tissues, leading to potential off-target toxicity when CAR-T cells target these antigens (40). Recent studies have focused on enhancing the specificity of CAR-T cell therapy to minimize off-tumor toxicity. One approach involves the use of synthetic Notch (SynNotch) receptors, which require the simultaneous recognition of two antigens to activate CAR-T cell cytotoxicity, thus increasing specificity for tumor cells (41). Another strategy employs inhibitory CARs (iCARs), which are engineered with inhibitory receptors that detect antigens expressed on normal tissues, preventing CAR-T cells from attacking healthy cells (42). CAR-NK cells also face the challenge of off-target toxicity due to antigens expressed on both tumors and normal tissues. SynNotch receptors, which require the recognition of two antigens to activate CAR-NK cell cytotoxicity, enhance specificity for tumor cells (43). Inhibitory CARs (iCARs) have also been developed for CAR-NK cells, helping prevent them from attacking healthy tissues (44). Together, these strategies improve the safety and precision of CAR-NK cell therapy in treating solid tumors.

### 2.5 Persistence and exhaustion of CAR-T and CAR-NK cells

CAR-T cells often face limited persistence and functional exhaustion within the hostile tumor microenvironment, diminishing their long-term efficacy against solid tumors (45). Recent advancements focus on enhancing CAR-T cell persistence and functionality. Engineering CAR-T cells to adopt a memory phenotype improves their longevity and anti-tumor activity (46). Additionally, metabolic reprogramming enhances CAR-T cell fitness, enabling them to thrive in nutrient-deprived and hypoxic conditions typical of solid tumors (47). Similar to CAR-T cells, CAR-NK cells can exhibit limited persistence and become exhausted within the tumor microenvironment. To address this, CAR-NK cells have been engineered to adopt a memory-like phenotype or to express cytokines like IL-15, improving their longevity and self-renewal capacity (48). Metabolic reprogramming ensures CAR-NK cells thrive in the harsh conditions of solid tumors (49). Optimizing co-stimulatory domains such as 2B4 or DAP12 and combining CAR-NK therapy with checkpoint inhibitors like PD-1 can help prevent exhaustion and sustain activity within the TME (50).

### 2.6 Antigen loss and tumor escape mechanisms

Tumors can develop resistance to CAR-T cell therapy by downregulating or mutating the target antigen, leading to relapse (51). Strategies like sequential targeting, where CAR-T cells targeting different antigens are administered in succession, have been proposed to reduce the likelihood of antigen escape (52). Universal CAR platforms that allow for quick retargeting to new antigens as tumor evolution occurs enhance therapy adaptability (53). Multi-targeted approaches and real-time antigen monitoring further improve the durability and efficacy of CAR-T cell therapy in solid tumors (54). Similarly, antigen loss or mutation can lead to resistance to CAR-NK cell therapy. Sequential targeting of different antigens or using universal CAR platforms to quickly retarget to evolving antigens can help overcome antigen escape (55, 56). Multi-targeted approaches and real-time antigen monitoring also ensure the continuous effectiveness of CAR-NK cell therapy against heterogeneous tumors (57).

### 2.7 Manufacturing and scalability issues

The manufacturing and scalability of CAR-T and CAR-NK cell therapies remain significant barriers to their widespread adoption, particularly for solid tumor indications that require sophisticated engineering (58). Both therapies face challenges related to the complexity and cost of personalized production. For CAR-T cells, the process involves isolating and genetically modifying a patient’s T cells, which is time-consuming, costly, and requires specialized facilities. This personalized approach limits the scalability of CAR-T therapy, making it difficult to expand its use, particularly in resource-constrained settings (59). In contrast, CAR-NK cells offer potential advantages in scalability. Recent studies have focused on improving the accessibility of CAR-NK therapy through innovative manufacturing approaches (60). One promising development is the creation of off-the-shelf CAR-NK cells, which are derived from healthy donors or induced pluripotent stem cells (iPSCs) (61). Unlike autologous CAR-NK cells, which require personalized production, off-the-shelf CAR-NK cells can be manufactured in bulk and stored for immediate use, significantly reducing production time and costs (62). This shift towards off-the-shelf CAR-NK cells enhances the efficiency and responsiveness of the therapy, making it more accessible and cost-effective compared to autologous CAR-T cell therapies. Furthermore, automated manufacturing platforms have been developed to improve scalability and consistency for both CAR-T and CAR-NK cells (63). These platforms utilize closed-system processes to minimize contamination risks, ensure high-quality products, and streamline workflows, making large-scale production more feasible. This is particularly important for CAR-NK cells, as the demand for off-the-shelf products could place considerable strain on production capabilities (64). By addressing the logistical and technical challenges of large-scale production, these innovations make both CAR-T and CAR-NK cell therapies more sustainable and accessible, particularly for treating solid tumors and other malignancies (65, 66).

### 2.8 Safety and toxicity: CRS and neurotoxicity in CAR-T vs. CAR-NK

CAR-T therapy has proven highly effective in treating hematologic malignancies[304]; however, it is associated with significant safety concerns, particularly cytokine release syndrome (CRS) and immune effector cell-associated neurotoxicity syndrome (ICANS) (67). CRS occurs as a result of massive cytokine release from activated T cells, primarily interleukin-6 (IL-6), and interferon-gamma (IFN-γ), which are critical in the pathophysiology of this adverse event (68). IL-6, in particular, plays a central role by driving fever, hypotension, and organ dysfunction (69), while IFN-γ can amplify the inflammatory response by inducing further cytokine release and immune cell activation (70). ICANS, which manifests as neurological toxicities such as encephalopathy, confusion, and seizures, is believed to be primarily triggered by the effects of cytokines on the blood-brain barrier and central nervous system (CNS) inflammation. To mitigate these risks, clinical management strategies have been developed. Tocilizumab, an IL-6 receptor antagonist, is commonly used to treat CRS by blocking the effects of IL-6, reducing the severity of the syndrome (71). Additionally, corticosteroids are often employed to manage severe cases of CRS and ICANS, by suppressing the inflammatory response and stabilizing the patient’s condition (72). In contrast, CAR-NK cell therapy, an emerging alternative to CAR-T, has demonstrated a reduced risk of both CRS and ICANS (73). This is largely attributed to the innate properties of NK cells, which have a transient persistence in the body and distinct cytokine profiles compared to T cells. NK cells generally produce lower levels of pro-inflammatory cytokines, such as IL-6, which significantly lowers the likelihood of CRS and neurotoxic effects (74). Furthermore, the shorter lifespan of NK cells *in vivo* means that they do not persist long enough to induce the prolonged cytokine-driven inflammation seen with CAR-T cells (75). As a result, CAR-NK therapy has been associated with fewer and less severe instances of both CRS and ICANS, offering a promising approach with a better safety profile in the treatment of solid tumors and hematologic malignancies (76). The reduced risk of CRS/ICANS with CAR-NK therapy makes it an attractive option, particularly for patients who may be at high risk for these toxicities with CAR-T cells (77). Ongoing clinical trials continue to explore ways to optimize CAR-NK cell therapy and further reduce safety concerns, making it a compelling alternative to traditional CAR-T therapies.

## 3 TCR-T cell therapy

TCR- T cell therapy is an innovative form of adoptive cell therapy that leverages the specificity of TCRs to recognize and target tumor antigens presented by Major Histocompatibility Complex (MHC) molecules (78). Unlike CAR-T cells, which recognize antigens in an MHC-independent manner, TCR-T cells can target intracellular antigens, thereby expanding the range of potential targets. This section explores the primary strategies for TCR-T cell therapy, including targeting tumor antigens, viral antigens, the KRAS gene, and immune checkpoints. Each subsection discusses the challenges, recent advancements, and potential solutions, supported by relevant studies from the past decade.

### 3.1 Targeting tumor antigens

Targeting tumor-specific antigens (TSAs) and tumor-associated antigens (TAAs) is central to TCR-T cell therapy. TSAs are unique to cancer cells, while TAAs are overexpressed in tumors. Challenges include antigen selection, MHC restriction, tumor heterogeneity, and immune tolerance to certain antigens, which limit the therapy’s efficacy (79). Recent advancements focus on improving specificity and efficacy. High-affinity TCRs, such as those targeting NY-ESO-1, have shown enhanced anti-tumor activity (80). Neoantigen targeting, where TCRs target tumor-specific mutations, has also shown promise (81). Universal TCRs that recognize multiple HLA types are being developed to expand treatment eligibility. Innovative strategies include multi-antigen targeting to overcome tumor heterogeneity and immune escape, and universal TCR platforms using CRISPR/Cas9 to create broadly applicable TCR-T cells. Personalized TCR-T therapy, enabled by next-generation sequencing (NGS), allows customization for individual patients, improving precision and effectiveness (82). These strategies collectively enhance the potential of TCR-T cell therapy in targeting a wide range of tumors with improved specificity and reduced risk of immune escape.

### 3.2 Targeting viral antigens

Targeting viral antigens with TCR-T cell therapy involves engineering TCR-T cells to recognize antigens from oncogenic viruses like Epstein-Barr Virus (EBV), Human Papillomavirus (HPV), and Hepatitis B Virus (HBV), which are linked to various cancers (83). Challenges include ensuring consistent high-level expression of viral antigens on tumor cells, immune evasion by viruses that downregulate antigen presentation or inhibit T cell function, and potential off-target effects on normal cells with latent infections (84). Additionally, the limited number of suitable antigens for TCR targeting restricts the range of targets for therapy. To improve efficacy and safety, several strategies are being explored. These include enhancing antigen presentation by combining TCR-T cell therapy with agents that upregulate MHC expression or block viral immune evasion (85). The development of dual-specific TCRs, which recognize both viral and tumor antigens, can enhance specificity and reduce immune escape (86). Safety mechanisms like inducible suicide genes can control TCR-T cell elimination in cases of severe toxicity, thereby reducing harm to normal tissues (87). These approaches aim to improve the precision, safety, and therapeutic potential of TCR-T cell therapies targeting viral antigens (88).

### 3.3 Targeting KRAS gene

KRAS is a frequently mutated oncogene in cancers such as pancreatic, colorectal, and lung cancers (89). Targeting KRAS mutations with TCR-T cell therapy offers promise, but challenges include mutation specificity, HLA restriction, tumor heterogeneity, and avoiding off-target effects on normal tissues (90). Designing TCRs that differentiate mutant from wild-type KRAS peptides and ensuring TCR-T cells target only tumor cells without causing toxicity are key issues. Recent studies show progress in KRAS-targeted TCR-T therapies. For example, TCR-T cells targeting KRAS G12D in pancreatic cancer have demonstrated preclinical efficacy (91). High-affinity TCRs for the KRAS G12V mutation have also shown improved anti-tumor activity with fewer off-target effects (92). Combining KRAS-targeted TCR-T cells with MEK inhibitors has exhibited synergistic effects, addressing resistance mechanisms in KRAS-mutant tumors. Potential solutions to enhance KRAS-targeted TCR-T therapy include multi-antigen targeting to address tumor heterogeneity, universal TCR platforms for broader patient applicability, and advanced gene editing techniques like CRISPR/Cas9 to improve TCR specificity and reduce off-target effects. These strategies could significantly improve the precision, efficacy, and scalability of KRAS-targeted therapies (93).

### 3.4 Targeting immune checkpoints

Targeting immune checkpoints in TCR-T cell therapy aims to enhance anti-tumor activity by modulating inhibitory pathways within the tumor microenvironment. Challenges include the need to target multiple checkpoints simultaneously, safety concerns such as autoimmunity, and tumor resistance through alternative inhibitory pathways (94). Persistent antigen exposure can also lead to T cell exhaustion, reducing therapeutic efficacy. Recent advancements focus on overcoming these challenges. Strategies include engineering TCR-T cells to secrete PD-1 blocking antibodies or express dominant-negative PD-1 receptors, thereby preventing exhaustion (95). Combining TCR-T therapy with CTLA-4 blockade or dual checkpoint blockade (e.g., PD-1 and TIM-3) has shown improved efficacy (96). Armored TCR-T cells expressing checkpoint inhibitors or pro-inflammatory cytokines like IL-12 create a more favorable tumor environment (97). Potential solutions include engineering armored TCR-T cells, employing synthetic biology approaches to modulate checkpoint pathways, and utilizing combination therapies with other immunotherapies, such as checkpoint inhibitors or cytokine therapies. These strategies enhance anti-tumor responses and efficacy, making TCR-T cell therapies more robust and effective against immune-evasive tumors (98).

## 4 Other immunotherapy approaches

These alternative immunotherapies—Tumor-Infiltrating Lymphocyte (TIL), Natural Killer (NK), and Cytokine-Induced Killer (CIK) cell therapies—are promising strategies in the treatment of various malignancies (99). While TIL therapy harnesses tumor-specific T cells to directly target cancer, NK cell therapy benefits from the innate immune system’s ability to recognize and kill tumors without MHC restriction. CIK cell therapy combines the properties of both T cells and NK cells, showing broad potential in eliminating a variety of cancers (100). However, challenges such as enhancing cell persistence, overcoming immunosuppressive microenvironments, and optimizing treatment protocols remain areas for further research and development.

### 4.1 Tumor-infiltrating lymphocyte therapy

Tumor-Infiltrating Lymphocyte (TIL) therapy is an adoptive cell therapy where T cells are isolated from a patient’s tumor, expanded ex vivo, and reinfused to attack cancer cells (101). TILs, especially CD8+ cytotoxic T lymphocytes (CTLs), recognize tumor antigens on cancer cell surfaces. TIL therapy has shown promising results in melanoma, with durable responses in patients who did not respond to conventional therapies (102). The process involves extracting tumor tissue, isolating TILs, expanding them with agents like interleukin-2 (IL-2), and reinfusing them to enhance the immune response against cancer (103). While effective in melanoma, challenges persist in improving TIL persistence and efficacy in other solid tumors due to the immunosuppressive tumor microenvironment (104). Recent studies have explored gene-editing techniques to enhance TIL function, highlighting the potential and the need for further advancements to optimize TIL therapy’s clinical outcomes (105). Future directions include enhancing TIL survival, improving their infiltration into tumors, and combining TIL therapy with other immunomodulatory treatments to overcome resistance mechanisms (106).

### 4.2 NK cell therapy

NK cells are innate immune cells capable of recognizing and killing cancer cells without prior sensitization (107). Unlike T cells, NK cells do not require antigen presentation via MHC molecules, enabling them to target a wider range of tumors, including those with low or absent MHC expression (108). NK cell therapy involves expanding and activating NK cells ex vivo, followed by reinfusion to target cancer cells (109). It has been explored for various cancers, including hematological malignancies (e.g., leukemia, lymphoma) and solid tumors (e.g., non-small cell lung cancer, ovarian cancer) (110).

A key challenge in NK cell therapy is enhancing their persistence and function within the immunosuppressive tumor microenvironment (111). Strategies to overcome this include genetic modification to enhance NK cell activity, cytokine support (e.g., IL-15), and combining NK cell therapy with other immune therapies like checkpoint inhibitors (112). Recent studies have shown progress in NK cell therapy. Rubio et al. demonstrated that NK cells engineered with a chimeric antigen receptor (CAR) targeting CD19 exhibited enhanced anti-tumor activity in B-cell malignancies (113). Additionally, Miller et al. reported promising results in NK cell therapy for acute myeloid leukemia (AML), highlighting its growing potential in cancer immunotherapy (114). Future advancements focus on improving NK cell persistence, enhancing their cytotoxicity, and integrating NK cell therapies with other treatment modalities to maximize therapeutic efficacy (115).

### 4.3 CIK cell therapy

CIK cell therapy is an adoptive immunotherapy approach where T cells are expanded with cytokines, such as IL-2 and IFN-γ, to generate highly cytotoxic lymphocytes capable of targeting and killing tumor cells (116). CIK cells are a heterogeneous population, including T cells and NK-like cells, with potent anti-tumor effects (117). They can recognize and eliminate tumor cells without antigen-specific activation, showing promise in treating both hematologic and solid tumors, such as non-small cell lung cancer, hepatocellular carcinoma, and colorectal cancer (118). However, challenges remain in optimizing cell expansion, enhancing persistence, and overcoming the immunosuppressive tumor microenvironment (119). Recent studies highlight the potential of CIK therapy. CIK cells, combined with chemotherapy, improved survival in advanced non-small cell lung cancer patients (120). Future research aims to enhance CIK cell proliferation, improve their trafficking to tumor sites, and integrate CIK therapy with other immunomodulatory treatments to enhance their anti-tumor efficacy.

## 5 The status of immunotherapy for cell-based treatment

### 5.1 Challenges

Despite significant progress, cell-based immunotherapies face numerous challenges. Manufacturing and scalability remain major obstacles, as personalized therapies like CAR-T and TCR-T cells are complex and costly to produce. This necessitates the development of universal cell platforms and automated manufacturing processes for broader scalability. The immunosuppressive TME poses another significant hurdle, requiring innovative strategies such as engineering cells to secrete pro-inflammatory cytokines, express dominant-negative receptors, and combine with checkpoint inhibitors to enhance their efficacy. Safety and toxicity issues, including managing on-target, off-tumor effects and severe toxicities like CRS and neurotoxicity, are crucial concerns that necessitate the incorporation of safety switches and improved target specificity. Additionally, resistance mechanisms present ongoing challenges, as tumors can develop resistance through antigen loss, upregulation of alternative checkpoints, and metabolic adaptations. This drives the exploration of multi-targeted and combination therapies to counteract these adaptive strategies. Addressing these multifaceted challenges is essential for the continued advancement and widespread adoption of cell-based immunotherapies in cancer treatment.

### 5.2 Future prospects

The future of cell-based immunotherapy is highly promising, driven by ongoing research aimed at enhancing specificity and efficacy through advanced gene editing, multi-antigen targeting, and synthetic biology approaches. Efforts are also focused on expanding the applications of these therapies to encompass a broader range of solid tumors and other malignancies, thereby increasing their therapeutic impact across diverse cancer types. Improving accessibility is another critical objective, with the development of off-the-shelf products and strategies to reduce manufacturing costs making these advanced treatments more widely available to patients. Furthermore, the integration of personalized medicine, leveraging genomic and proteomic data, allows for the tailoring of therapies to individual patient profiles, thereby enhancing treatment outcomes and ensuring more precise and effective cancer management. These advancements collectively pave the way for more robust, versatile, and patient-centric cell-based immunotherapies in the fight against cancer.

## 6 Conclusion

Cell-based immunotherapies, including CAR-T, CAR-NK, and TCR-T therapies, demonstrate immense potential in cancer treatment, particularly excelling in hematologic malignancies with significant clinical successes. These therapies enhance treatment efficacy by precisely targeting and eliminating tumor cells. However, their application in solid tumors faces several challenges, such as antigen heterogeneity, immunosuppressive tumor microenvironments, limited cell infiltration, off-target toxicity, cell persistence, and manufacturing scalability. Despite ongoing challenges, the future of cell-based immunotherapies in cancer treatment remains promising. Continued research and innovation are crucial to overcoming issues related to safety, efficacy, and scalability, ultimately providing more precise and personalized treatment options for cancer patients. By addressing these challenges, cell-based immunotherapies can fulfill their potential in transforming cancer care and improving patient outcomes on a global scale.

## References

[1] Siegel RL Miller KD Wagle NS Jemal A. Cancer statistics, 2023. CA Cancer J Clin. (2023) 73(1):17–48. doi: 10.3322/caac.21763 36633525

[2] Hanahan D Weinberg RA. Hallmarks of cancer: the next generation. Cell. (2011) 144(5):646–74. doi: 10.1016/j.cell.2011.02.013 21376230

[3] June CH O'Connor RS Kawalekar OU Ghassemi S Milone MC. CAR t cell immunotherapy for human cancer. Science. (2018) 359(6382):1361–5. doi: 10.1126/science.aar6711 29567707

[4] Rosenberg SA Restifo NP Yang JC Morgan RA Dudley ME. Adoptive cell transfer: a clinical path to effective cancer immunotherapy. Nat Rev Cancer. (2008) 8(4):299–308. doi: 10.1038/nrc2355 18354418 PMC2553205

[5] June CH Sadelain M. Chimeric antigen receptor therapy. N Engl J Med. (2018) 379(1):64–73. doi: 10.1056/NEJMra1706169 29972754 PMC7433347

[6] Maude SL Laetsch TW Buechner J Rives S Boyer M Bittencourt H. Tisagenlecleucel in children and young adults with b-cell lymphoblastic leukemia. N Engl J Med. (2018) 378(5):439–48. doi: 10.1056/NEJMoa1709866 PMC5996391 29385370

[7] Brudno JN Maus MV Hinrichs CS Cells CART. And t-cell therapies for cancer: A translational science review. JAMA. (2024) 332(22):1924–35. doi: 10.1001/jama.2024.19462 PMC11808657 39495525

[8] Neelapu SS Locke FL Bartlett NL Lekakis LJ Miklos DB Jacobson CA. Axicabtagene ciloleucel CAR t-cell therapy in refractory large b-cell lymphoma. N Engl J Med. (2017) 377(26):2531–44. doi: 10.1056/NEJMoa1707447 PMC5882485 29226797

[9] Page A Chuvin N Valladeau-Guilemond J Depil S. Development of NK cell-based cancer immunotherapies through receptor engineering. Cell Mol Immunol. (2024) 21(4):315–31. doi: 10.1038/s41423-024-01145-x PMC10978891 38443448

[10] Lee DW Santomasso BD Locke FL Ghobadi A Turtle CJ Brudno JN. ASTCT consensus grading for cytokine release syndrome and neurologic toxicity associated with immune effector cells. Biol Blood Marrow Transplant. (2019) 25(4):625–38. doi: 10.1016/j.bbmt.2018.12.758 PMC12180426 30592986

[11] Hirabayashi K Du H Xu Y Shou P Zhou X Fucá G. Dual targeting CAR-t cells with optimal costimulation and metabolic fitness enhance antitumor activity and prevent escape in solid tumors. Nat Cancer. (2021) 2(9):904–18. doi: 10.1038/s43018-021-00244-2 PMC8570569 34746799

[12] Hegde M Mukherjee M Grada Z Pignata A Landi D Navai SA. Tandem CAR t cells targeting HER2 and IL13Rα2 mitigate tumor antigen escape. J Clin Invest. (2016) 126(8):3036–52. doi: 10.1172/JCI83416 PMC4966331 27427982

[13] Schmidts A Srivastava AA Ramapriyan R Bailey SR Bouffard AA Cahill DP. Tandem chimeric antigen receptor (CAR) t cells targeting EGFRvIII and IL-13Rα2 are effective against heterogeneous glioblastoma. Neurooncol Adv. (2022) 5(1):vdac185. doi: 10.1093/noajnl/vdac185 36751672 PMC9896600

[14] Wang W Liu Y He Z Li L Liu S Jiang M. Breakthrough of solid tumor treatment: CAR-NK immunotherapy. Cell Death Discovery. (2024) 10(1):40. doi: 10.1038/s41420-024-01815-9 38245520 PMC10799930

[15] Campion S Aubrecht J Boekelheide K Brewster DW Vaidya VS Anderson L. The current status of biomarkers for predicting toxicity. Expert Opin Drug Metab Toxicol. (2013) 9(11):1391–408. doi: 10.1517/17425255.2013.827170 PMC3870154 23961847

[16] Fujiwara K Masutani M Tachibana M Okada N. Impact of scFv structure in chimeric antigen receptor on receptor expression efficiency and antigen recognition properties. Biochem Biophys Res Commun. (2020) 527(2):350–7. doi: 10.1016/j.bbrc.2020.03.071 32216966

[17] Duan Y Chen R Huang Y Meng X Chen J Liao C. Tuning the ignition of CAR: optimizing the affinity of scFv to improve CAR-t therapy. Cell Mol Life Sci. (2021) 79(1):14. doi: 10.1007/s00018-021-04089-x 34966954 PMC11073403

[18] Roselli E Boucher JC Li G Kotani H Spitler K Reid K. 4-1BB and optimized CD28 co-stimulation enhances function of human mono-specific and bi-specific third-generation CAR t cells. J Immunother Cancer. (2021) 9(10):e003354. doi: 10.1136/jitc-2021-003354 34706886 PMC8552146

[19] Nayak S Herzog RW. Progress and prospects: immune responses to viral vectors. Gene Ther. (2010) 17(3):295–304. doi: 10.1038/gt.2009.148 19907498 PMC3044498

[20] Gurumoorthy N Nordin F Tye GJ Wan Kamarul Zaman WS Ng MH. Non-integrating lentiviral vectors in clinical applications: A glance through. Biomedicines. (2022) 10(1):107. doi: 10.3390/biomedicines10010107 35052787 PMC8773317

[21] Sherba JJ Hogquist S Lin H Shan JW Shreiber DI Zahn JD. The effects of electroporation buffer composition on cell viability and electro-transfection efficiency. Sci Rep. (2020) 10(1):3053. doi: 10.1038/s41598-020-59790-x 32080269 PMC7033148

[22] Tran E Robbins PF Lu YC Prickett TD Gartner JJ Jia L. T-cell transfer therapy targeting mutant KRAS in cancer. N Engl J Med. (2016) 375(23):2255–62. doi: 10.1056/NEJMoa1609279 PMC5178827 27959684

[23] Yeku OO Purdon TJ Koneru M Spriggs D Brentjens RJ. Armored CAR t cells enhance antitumor efficacy and overcome the tumor microenvironment. Sci Rep. (2017) 7(1):10541. doi: 10.1038/s41598-017-10940-8 28874817 PMC5585170

[24] Grosser R Cherkassky L Chintala N Adusumilli PS. Combination immunotherapy with CAR t cells and checkpoint blockade for the treatment of solid tumors. Cancer Cell. (2019) 36(5):471–82. doi: 10.1016/j.ccell.2019.09.006 PMC7171534 31715131

[25] Pan S Wang F Jiang J Lin Z Chen Z Cao T. Chimeric antigen receptor-natural killer cells: A new breakthrough in the treatment of solid tumours. Clin Oncol (R Coll Radiol). (2023) 35(3):153–62. doi: 10.1016/j.clon.2022.10.019 36437159

[26] ZZhao D Zhu D Cai F Jiang M Liu X Li T. Current situation and prospect of adoptive cellular immunotherapy for malignancies. Technol Cancer Res Treat. (2023) 22:15330338231204198. doi: 10.1177/15330338231204198 38037341 PMC10693217

[27] Li J Hu H Lian K Zhang D Hu P He Z. CAR-NK cells in combination therapy against cancer: A potential paradigm. Heliyon. (2024) 10(5):e27196. doi: 10.1016/j.heliyon.2024.e27196 38486782 PMC10937699

[28] Mehta RS Rezvani K. Chimeric antigen receptor expressing natural killer cells for the immunotherapy of cancer. Front Immunol. (2018) 9:283. doi: 10.3389/fimmu.2018.00283 29497427 PMC5818392

[29] Schluns KS Lefrançois L. Cytokine control of memory t-cell development and survival. Nat Rev Immunol. (2003) 3(4):269–79. doi: 10.1038/nri1052 12669018

[30] Mathieu C Beltra JC Charpentier T Bourbonnais S Di Santo JP Lamarre A. IL-2 and IL-15 regulate CD8+ memory t-cell differentiation but are dispensable for protective recall responses. Eur J Immunol. (2015) 45(12):3324–38. doi: 10.1002/eji.201546000 26426795

[31] Cao P Sun Z Zhang F Zhang J Zheng X Yu B. TGF-β enhances immunosuppression of myeloid-derived suppressor cells to induce transplant immune tolerance through affecting arg-1 expression. Front Immunol. (2022) 13:919674. doi: 10.3389/fimmu.2022.919674 35874674 PMC9300822

[32] Yeku OO Brentjens RJ. Armored CAR t-cells: utilizing cytokines and pro-inflammatory ligands to enhance CAR t-cell anti-tumour efficacy. Biochem Soc Trans. (2016) 44(2):412–8. doi: 10.1042/BST20150291 PMC5529098 27068948

[33] Brummel K Eerkens AL de Bruyn M Nijman HW. Tumour-infiltrating lymphocytes: from prognosis to treatment selection. Br J Cancer. (2023) 128(3):451–8. doi: 10.1038/s41416-022-02119-4 PMC9938191 36564565

[34] Sterner RC Sterner RM. CAR-t cell therapy: current limitations and potential strategies. Blood Cancer J. (2021) 11(4):69. doi: 10.1038/s41408-021-00459-7 33824268 PMC8024391

[35] Ikeda-Imafuku M Gao Y Shaha S Wang LL Park KS Nakajima M. Extracellular matrix degrading enzyme with stroma-targeting peptides enhance the penetration of liposomes into tumors. J Control Release. (2022) 352:1093–103. doi: 10.1016/j.jconrel.2022.11.007 36351520

[36] Uccelli A Moretta L Pistoia V. Mesenchymal stem cells in health and disease. Nat Rev Immunol. (2008) 8(9):726–36. doi: 10.1038/nri2395 19172693

[37] Ran GH Lin YQ Tian L Zhang T Yan DM Yu JH. Natural killer cell homing and trafficking in tissues and tumors: from biology to application. Signal Transduct Target Ther. (2022) 7(1):205. doi: 10.1038/s41392-022-01058-z 35768424 PMC9243142

[38] Qin Y Xu G. Enhancing CAR t-cell therapies against solid tumors: Mechanisms and reversion of resistance. Front Immunol. (2022) 13:1053120. doi: 10.3389/fimmu.2022.1053120 36569859 PMC9773088

[39] Schmeel LC Schmeel FC Coch C Schmidt-Wolf IG. Cytokine-induced killer (CIK) cells in cancer immunotherapy: report of the international registry on CIK cells (IRCC). J Cancer Res Clin Oncol. (2015) 141(5):839–49. doi: 10.1007/s00432-014-1864-3 PMC11824144 25381063

[40] Choe JH Watchmaker PB Simic MS Gilbert RD Li AW Krasnow NA. SynNotch-CAR t cells overcome challenges of specificity, heterogeneity, and persistence in treating glioblastoma. Sci Transl Med. (2021) 13(591):eabe7378. doi: 10.1126/scitranslmed.abe7378 33910979 PMC8362330

[41] Funk MA Heller G Waidhofer-Söllner P Leitner J Steinberger P. Inhibitory CARs fail to protect from immediate t cell cytotoxicity. Mol Ther. (2024) 32(4):982–99. doi: 10.1016/j.ymthe.2024.02.022 PMC11163222 38384128

[42] Zhu B Yin H Zhang D Zhang M Chao X Scimeca L. Synthetic biology approaches for improving the specificity and efficacy of cancer immunotherapy. Cell Mol Immunol. (2024) 21(5):436–47. doi: 10.1038/s41423-024-01153-x PMC11061174 38605087

[43] D'Aloia MM Zizzari IG Sacchetti B Pierelli L Alimandi M. CAR-t cells: the long and winding road to solid tumors. Cell Death Dis. (2018) 9(3):282. doi: 10.1038/s41419-018-0278-6 29449531 PMC5833816

[44] Morgan RA Yang JC Kitano M Dudley ME Laurencot CM Rosenberg SA. Case report of a serious adverse event following the administration of t cells transduced with a chimeric antigen receptor recognizing ERBB2. Mol Ther. (2010) 18(4):843–51. doi: 10.1038/mt.2010.24 PMC2862534 20179677

[45] Syed F El Fakih R Alahmari AD Osman Ali AS Aljurf M. Chimeric antigen receptor structure and manufacturing of clinical grade CAR engineered cells using different bioreactors. Hematol Oncol Stem Cell Ther. (2022) 15(3):137–52. doi: 10.56875/2589-0646.1048 36395497

[46] Shi Y Kotchetkov IS Dobrin A Hanina SA Rajasekhar VK Healey JH. GLUT1 overexpression enhances CAR t cell metabolic fitness and anti-tumor efficacy. Mol Ther. (2024) 32(7):2393–405. doi: 10.1016/j.ymthe.2024.05.006 PMC11286825 38720457

[47] Marofi F Rahman HS Thangavelu L Dorofeev A Bayas-Morejón F Shirafkan N. Renaissance of armored immune effector cells, CAR-NK cells, brings the higher hope for successful cancer therapy. Stem Cell Res Ther. (2021) 12(1):200. doi: 10.1186/s13287-021-02251-7 33752707 PMC7983395

[48] Chen R Li L Feng L Luo Y Xu M Leong KW. Biomaterial-assisted scalable cell production for cell therapy. Biomaterials. (2020) 230:119627. doi: 10.1016/j.biomaterials.2019.119627 31767445

[49] Siegler EL Zhu Y Wang P Yang L. Off-the-Shelf CAR-NK cells for cancer immunotherapy. Cell Stem Cell. (2018) 23(2):160–1. doi: 10.1016/j.stem.2018.07.007 30075127

[50] Chen C Wang Z Qin Y. CRISPR/Cas9 system: recent applications in immuno-oncology and cancer immunotherapy. Exp Hematol Oncol. (2023) 12(1):95. doi: 10.1186/s40164-023-00457-4 37964355 PMC10647168

[51] Lin H Yang X Ye S Huang L Mu W. Antigen escape in CAR-t cell therapy: Mechanisms and overcoming strategies. BioMed Pharmacother. (2024), 178:117252. doi: 10.1016/j.biopha.2024.117252 39098176

[52] Devaney A Carton J Hoffman H Millar HJ Walker D Wheeler J. A novel, universal targeting receptor-adaptor CAR platform demonstrates versatile, flexible, and controlled tumor inhibition in an ipsc-derived t-cell. Blood. (2024) 144(Supplement 1):7181.

[53] Ma S Li X Wang X Cheng L Li Z Zhang C. Current progress in CAR-t cell therapy for solid tumors. Int J Biol Sci. (2019) 15(12):2548–60. doi: 10.7150/ijbs.34213 PMC6854376 31754328

[54] Eitler J Rackwitz W Wotschel N Gudipati V Murali Shankar N Sidorenkova A. CAR-mediated targeting of NK cells overcomes tumor immune escape caused by ICAM-1 downregulation. J Immunother Cancer. (2024) 12(2):e008155. doi: 10.1136/jitc-2023-008155 38417916 PMC10900364

[55] Cho JH Collins JJ Wong WW. Universal chimeric antigen receptors for multiplexed and logical control of t cell responses. Cell. (2018) 173(6):1426–38. doi: 10.1016/j.cell.2018.03.038 PMC5984158 29706540

[56] Mohammad A Yurina A Simonyan T Chistyakov D Salman R Zornikova K. Modular (universal) CAR-t platforms *in vivo*: a comprehensive systematic review. Front Immunol. (2024) 15:1409665. doi: 10.3389/fimmu.2024.1409665 39712013 PMC11659234

[57] Chan LY Dass SA Tye GJ Imran SAM Wan Kamarul Zaman WS Nordin F. CAR-t cells/-NK cells in cancer immunotherapy and the potential of MSC to enhance its efficacy: A review. Biomedicines. (2022) 10(4):804. doi: 10.3390/biomedicines10040804 35453554 PMC9024487

[58] Albinger N Müller S Kostyra J Kuska J Mertlitz S Penack O. Manufacturing of primary CAR-NK cells in an automated system for the treatment of acute myeloid leukemia. Bone Marrow Transplant. (2024) 59(4):489–95. doi: 10.1038/s41409-023-02180-4 PMC10994833 38253870

[59] Basar R Daher M Rezvani K. Next-generation cell therapies: the emerging role of CAR-NK cells. Blood Adv. (2020) 4(22):5868–76. doi: 10.1182/bloodadvances.2020002547 PMC7686910 33232480

[60] Daher M Melo Garcia L Li Y Rezvani K. CAR-NK cells: the next wave of cellular therapy for cancer. Clin Transl Immunol. (2021) 10(4):e1274. doi: 10.1002/cti2.1274 PMC8080297 33959279

[61] Lin X Sun Y Dong X Liu Z Sugimura R Xie G. IPSC-derived CAR-NK cells for cancer immunotherapy. BioMed Pharmacother. (2023), 165:115123. doi: 10.1016/j.biopha.2023.115123 37406511

[62] Xie G Dong H Liang Y Ham JD Rizwan R Chen J. CAR-NK cells: A promising cellular immunotherapy for cancer. EBioMedicine. (2020), 59:102975. doi: 10.1016/j.ebiom.2020.102975 PMC7452675 32853984

[63] Smith D Heathman TRJ Klarer A LeBlon C Tada Y Hampson B. Towards automated manufacturing for cell therapies. Curr Hematol Malig Rep. (2019) 14(4):278–85. doi: 10.1007/s11899-019-00522-y 31254154

[64] Morgan MA Büning H Sauer M Schambach A. Use of cell and genome modification technologies to generate improved "Off-the-Shelf" CAR t and CAR NK cells. Front Immunol. (2020) 11:1965. doi: 10.3389/fimmu.2020.01965 32903482 PMC7438733

[65] Lu Y Xu X Wang L. Smart manufacturing process and system automation – a critical review of the standards and envisioned scenarios. J Manufacturing Syst. (2020) 56:312–25. doi: 10.1016/j.jmsy.2020.06.010

[66] Peng L Sferruzza G Yang L Zhou L Chen S. CAR-t and CAR-NK as cellular cancer immunotherapy for solid tumors. Cell Mol Immunol. (2024) 21(10):1089–108. doi: 10.1038/s41423-024-01207-0 PMC11442786 39134804

[67] Freyer CW Porter DL. Cytokine release syndrome and neurotoxicity following CAR t-cell therapy for hematologic malignancies. J Allergy Clin Immunol. (2020) 146(5):940–8. doi: 10.1016/j.jaci.2020.07.025 32771558

[68] Cobb DA Lee DW. Cytokine release syndrome biology and management. Cancer J. (2021) 27(2):119–25. doi: 10.1097/PPO.0000000000000515 33750071

[69] Aliyu M Zohora FT Anka AU Ali K Maleknia S Saffarioun M. Interleukin-6 cytokine: An overview of the immune regulation, immune dysregulation, and therapeutic approach. Int Immunopharmacol. (2022) 111:109130. doi: 10.1016/j.intimp.2022.109130 35969896

[70] Ivashkiv LB. IFNγ: signalling, epigenetics and roles in immunity, metabolism, disease and cancer immunotherapy. Nat Rev Immunol. (2018) 18(9):545–58. doi: 10.1038/s41577-018-0029-z PMC6340644 29921905

[71] Sebba A. Tocilizumab: the first interleukin-6-receptor inhibitor. Am J Health Syst Pharm. (2008) 65(15):1413–8. doi: 10.2146/ajhp070449 18653811

[72] Lakomy T Akhoundova D Nilius H Kronig MN Novak U Daskalakis M. Early use of corticosteroids following CAR t-cell therapy correlates with reduced risk of high-grade CRS without negative impact on neurotoxicity or treatment outcome. Biomolecules. (2023) 13(2):382. doi: 10.3390/biom13020382 36830750 PMC9953517

[73] Cienfuegos-Jimenez O Vazquez-Garza E Rojas-Martinez A. CAR-NK cells for cancer therapy: Molecular redesign of the innate antineoplastic response. Curr Gene Ther. (2022) 22(4):303–18. doi: 10.2174/1566523222666211217091724 34923939

[74] Pro- and anti-inflammatory cytokines in the context of NK cell-trophoblast interactions. Int J Mol Sci. (2022) 23(4):2387. 35216502 10.3390/ijms23042387 PMC8878424

[75] Fang F Xie S Chen M Li Y Yue J Ma J. Advances in NK cell production. Cell Mol Immunol. (2022) 19(4):460–81. doi: 10.1038/s41423-021-00808-3 PMC8975878 34983953

[76] HosseinalizadehHWangLSMirzaeiHAmoozgarZTianLYuJ. Emerging combined CAR-NK cell therapies in cancer treatment: Finding a dancing partner. Mol Ther. (2025) 3:S1525–0016(24)00895-5. doi: 10.1016/j.ymthe.2024.12.057 39754357

[77] Xiao X Huang S Chen S Wang Y Sun Q Xu X. Mechanisms of cytokine release syndrome and neurotoxicity of CAR t-cell therapy and associated prevention and management strategies. J Exp Clin Cancer Res. (2021) 40(1):367. doi: 10.1186/s13046-021-02148-6 34794490 PMC8600921

[78] Tsimberidou AM Van Morris K Vo HH Eck S Lin YF Rivas JM. T-cell receptor-based therapy: an innovative therapeutic approach for solid tumors. J Hematol Oncol. (2021) 14(1):102. doi: 10.1186/s13045-021-01115-0 34193217 PMC8243554

[79] Arndt C Fasslrinner F Loureiro LR Koristka S Feldmann A Bachmann M. Adaptor CAR platforms-next generation of t cell-based cancer immunotherapy. Cancers (Basel). (2020) 12(5):1302. doi: 10.3390/cancers12051302 32455621 PMC7281723

[80] Ali A DiPersio JF. ReCARving the future: bridging CAR t-cell therapy gaps with synthetic biology, engineering, and economic insights. Front Immunol. (2024), 15:1432799. doi: 10.3389/fimmu.2024.1432799 PMC11410633 39301026

[81] Füchsl F Untch J Kavaka V Zuleger G Braun S Schwanzer A. High-resolution profile of neoantigen-specific TCR activation links moderate stimulation to increased resilience of engineered TCR-t cells. Nat Commun. (2024) 15(1):10520. doi: 10.1038/s41467-024-53911-0 39627205 PMC11615276

[82] Pang Z Lu MM Zhang Y Gao Y Bai JJ Gu JY. Neoantigen-targeted TCR-engineered t cell immunotherapy: current advances and challenges. biomark Res. (2023) 11(1):104. doi: 10.1186/s40364-023-00534-0 38037114 PMC10690996

[83] Tian Y Xie D Yang L. Engineering strategies to enhance oncolytic viruses in cancer immunotherapy. Signal Transduct Target Ther. (2022) 7(1):117. doi: 10.1038/s41392-022-00951-x 35387984 PMC8987060

[84] Sharma P Allison JP. Immune checkpoint targeting in cancer therapy: toward combination strategies with curative potential. Cell. (2015) 161(2):205–14. doi: 10.1016/j.cell.2015.03.030 PMC5905674 25860605

[85] Ruella M Korell F Porazzi P Maus MV. Mechanisms of resistance to chimeric antigen receptor-t cells in haematological malignancies. Nat Rev Drug Discovery. (2023) 22(12):976–95. doi: 10.1038/s41573-023-00807-1 PMC10965011 37907724

[86] Niu X Zhang P Dai L Peng X Liu Z Tang Y. Flagellin engineering enhances CAR-t cell function by reshaping tumor microenvironment in solid tumors. J Immunother Cancer. (2025) 13(4):e010237. doi: 10.1136/jitc-2024-010237 40187752 PMC11973770

[87] Yan T Zhu L Chen J. Current advances and challenges in CAR t-cell therapy for solid tumors: tumor-associated antigens and the tumor microenvironment. Exp Hematol Oncol. (2023) 12(1):14. doi: 10.1186/s40164-023-00373-7 36707873 PMC9883880

[88] Karschnia P Teske N Thon N Subklewe M Tonn JC Dietrich J. Chimeric antigen receptor t cells for glioblastoma: Current concepts, challenges, and future perspectives. Neurology. (2021) 97(5):218–30. doi: 10.1212/WNL.0000000000012193 33986138

[89] Poole A Karuppiah V Hartt A Haidar JN Moureau S Dobrzycki T. Therapeutic high affinity t cell receptor targeting a KRASG12D cancer neoantigen. Nat Commun. (2022) 13(1):5333. doi: 10.1038/s41467-022-32811-1 36088370 PMC9464187

[90] Zhang M Xu W Luo L Guan F Wang X Zhu P. Identification and affinity enhancement of t-cell receptor targeting a KRASG12V cancer neoantigen. Commun Biol. (2024) 7(1):512. doi: 10.1038/s42003-024-06209-2 38684865 PMC11058820

[91] Leidner R Sanjuan Silva N Huang H Sprott D Zheng C. Neoantigen t-cell receptor gene therapy in pancreatic cancer. N Engl J Med. (2022) 386(22):2112–9. doi: 10.1056/NEJMoa2119662 PMC9531755 35648703

[92] Lu D Chen Y Jiang M Wang J Li Y Ma K. KRAS G12V neoantigen specific t cell receptor for adoptive t cell therapy against tumors. Nat Commun. (2023) 14(1):6389. doi: 10.1038/s41467-023-42010-1 37828002 PMC10570350

[93] Yang B Li X Fu Y Guo E Ye Y Li F. MEK inhibition remodels the immune landscape of mutant KRAS tumors to overcome resistance to PARP and immune checkpoint inhibitors. Cancer Res. (2021) 81(10):2714–29. doi: 10.1158/0008-5472.CAN-20-2370 PMC8265237 33589518

[94] Pardoll DM. The blockade of immune checkpoints in cancer immunotherapy. Nat Rev Cancer. (2012) 12(4):252–64. doi: 10.1038/nrc3239 PMC4856023 22437870

[95] Rath JA Arber C. Engineering strategies to enhance TCR-based adoptive t cell therapy. Cells. (2020) 9(6):1485. doi: 10.3390/cells9061485 32570906 PMC7349724

[96] Winge-Main AK Wälchli S Inderberg EM. T cell receptor therapy against melanoma-immunotherapy for the future? Scand J Immunol 2020 92(4):e12927. doi: 10.1111/sji.12927 32640053

[97] Kalos M Levine BL Porter DL Katz S Grupp SA Bagg A. T cells with chimeric antigen receptors have potent antitumor effects and can establish memory in patients with advanced leukemia. Sci Transl Med. (2011) 3(95):95ra73. doi: 10.1126/scitranslmed.3002842 PMC3393096 21832238

[98] Kilgour MK Bastin DJ Lee SH Ardolino M McComb S Visram A. Advancements in CAR-NK therapy: lessons to be learned from CAR-t therapy. Front Immunol. (2023) 14:1166038. doi: 10.3389/fimmu.2023.1166038 37205115 PMC10187144

[99] Vivier E Rebuffet L Narni-Mancinelli E Cornen S Igarashi RY Fantin VR. Natural killer cell therapies. Nature. (2024) 626(8000):727–36. doi: 10.1038/s41586-023-06945-1 38383621

[100] Meng Y Yu Z Wu Y Du T Chen S Meng F. Cell-based immunotherapy with cytokine-induced killer (CIK) cells: From preparation and testing to clinical application. Hum Vaccin Immunother. (2017) 13(6):1–9. doi: 10.1080/21645515.2017.1285987 PMC5489295 28301281

[101] Andersen R Donia M Ellebaek E Borch TH Kongsted P Iversen TZ. Long-lasting complete responses in patients with metastatic melanoma after adoptive cell therapy with tumor-infiltrating lymphocytes and an attenuated IL2 regimen. Clin Cancer Res. (2016) 22(15):3734–45. doi: 10.1158/1078-0432.CCR-15-1879 27006492

[102] Betof Warner A Corrie PG Hamid O. Tumor-infiltrating lymphocyte therapy in melanoma: Facts to the future. Clin Cancer Res. (2023) 29(10):1835–54. doi: 10.1158/1078-0432.CCR-22-1922 PMC10183807 36485001

[103] Jiménez-Reinoso A Nehme-Álvarez D Domínguez-Alonso C Álvarez-Vallina L. Synthetic TILs: Engineered tumor-infiltrating lymphocytes with improved therapeutic potential. Front Oncol. (2021) 10:593848. 33680923 10.3389/fonc.2020.593848 PMC7928359

[104] Zhang L Meng Y Yao H Zhan R Chen S Miao W. CAR-NK cells for acute myeloid leukemia immunotherapy: past, present and future. Am J Cancer Res. (2023) 13(11):5559–76. PMC10695781 38058830

[105] Wang L Dai Y Zhu F Qiu Z Wang Y Hu Y. Efficacy of DC-CIK-based immunotherapy combined with chemotherapy in the treatment of intermediate to advanced non-small cell lung cancer. Am J Transl Res. (2021) 13(11):13076–83. PMC8661198 34956526

[106] Ma Y Xu YC Tang L Zhang Z Wang J Wang HX. Cytokine-induced killer (CIK) cell therapy for patients with hepatocellular carcinoma: efficacy and safety. Exp Hematol Oncol. (2012) 1(1):11. doi: 10.1186/2162-3619-1-11 23210562 PMC3514101

[107] He B Mai Q Pang Y Deng S He Y Xue R. Cytokines induced memory-like NK cells engineered to express CD19 CAR exhibit enhanced responses against b cell malignancies. Front Immunol. (2023) 14:1130442. doi: 10.3389/fimmu.2023.1130442 37207215 PMC10191231

[108] Russick J Torset C Hemery E Cremer I. NK cells in the tumor microenvironment: Prognostic and theranostic impact. Recent Adv trends. Semin Immunol. (2020) 48:101407. doi: 10.1016/j.smim.2020.101407 32900565

[109] Dwarshuis NJ Parratt K Santiago-Miranda A Roy K. Cells as advanced therapeutics: State-of-the-art, challenges, and opportunities in large scale biomanufacturing of high-quality cells for adoptive immunotherapies. Adv Drug Delivery Rev. (2017) 114:222–39. doi: 10.1016/j.addr.2017.06.005 28625827

[110] Heipertz EL Zynda ER Stav-Noraas TE Hungler AD Boucher SE Kaur N. Current perspectives on "Off-The-Shelf" allogeneic NK and CAR-NK cell therapies. Front Immunol. (2021) 12:732135. doi: 10.3389/fimmu.2021.732135 34925314 PMC8671166

[111] Choi E Chang J-W Krueger J Lahr WS Pomeroy E Walsh M. Engineering CD70-directed CAR-NK cells for the treatment of hematological and solid malignancies. Blood. (2021) 138(Supplement 1):1691.34324630

[112] Marofi F Abdul-Rasheed OF Rahman HS Budi HS Jalil AT Yumashev AV. CAR-NK cell in cancer immunotherapy; a promising frontier. Cancer Sci. (2021) 112(9):3427–36. doi: 10.1111/cas.14993 PMC8409419 34050690

[113] Khawar MB Sun H. CAR-NK cells: From natural basis to design for kill. Front Immunol. (2021) 12:707542. doi: 10.3389/fimmu.2021.707542 34970253 PMC8712563

[114] Mueller S Sohmen M Kostyra J Mekes A Nitsche M Luevano ME. GMP-compliant, automated process for generation of CAR NK cells in a closed system for clinical use. Cytotherapy. (2020) 22(Supplement 5):S205–6.

[115] Evgin L Kottke T Tonne J Thompson J Huff AL van Vloten J. Oncolytic virus-mediated expansion of dual-specific CAR t cells improves efficacy against solid tumors in mice. Sci Transl Med. (2022) 14(640):eabn2231. doi: 10.1126/scitranslmed.abn2231 35417192 PMC9297825

[116] Hontscha C Borck Y Zhou H Messmer D Schmidt-Wolf IG. Clinical trials on CIK cells: first report of the international registry on CIK cells (IRCC). J Cancer Res Clin Oncol. (2011) 137(2):305–10. doi: 10.1007/s00432-010-0887-7 PMC11828187 20407789

[117] Wu X Jiang J Gu Z Zhang J Chen Y Liu X. Mesenchymal stromal cell therapies: immunomodulatory properties and clinical progress. Stem Cell Res Ther. (2020) 11(1):345. doi: 10.1186/s13287-020-01855-9 32771052 PMC7414268

[118] Ma Y Zhang Z Tang L Xu YC Xie ZM Gu XF. Cytokine-induced killer cells in the treatment of patients with solid carcinomas: a systematic review and pooled analysis. Cytotherapy. (2012) 14(4):483–93. doi: 10.3109/14653249.2011.649185 22277010

[119] Chen H Wang F Zhang P Zhang Y Chen Y Fan X. Management of cytokine release syndrome related to CAR-t cell therapy. Front Med. (2019) 13(5):610–7. doi: 10.1007/s11684-019-0714-8 31571160

[120] Li R Wang C Liu L Du C Cao S Yu J. Autologous cytokine-induced killer cell immunotherapy in lung cancer: a phase II clinical study. Cancer Immunol Immunother. (2012) 61(11):2125–33. doi: 10.1007/s00262-012-1260-2.v PMC11028805 22581306

